# Supplementary material for: Comparison of EfficientNet CNN models for multi-label chest X-ray disease diagnosis
Source: PeerJ Comput Sci. 2025 Jul 1;11:e2968. doi: 10.7717/peerj-cs.2968 (PMC12453788; doi:10.7717/peerj-cs.2968)
Supplement: Supplemental Information 1 [file peerj-cs-11-2968-s001.docx]

**Installation & Usage**

The project was tested on a virtual environment of python 3.7, pip 23.2.1, and MacOS Silicon

To run the jupyter notebook, which contains the project:

Download a 64-bit version of Python

Open console at .ipynb location

Acquire necessary libraries: "pip install tensorflow keras scikit-learn scipy Pillow pandas matplotlib jupyter and etc."

Run "jupyter notebook" and open the Project

OR

Training and testing can be conducted using Google Colab Pro+, a paid version of Google Colab. When using the Google Colab version, it should not be forgotten to integrate the dataset into the worksheet.

**Dataset**

The following information was supplied regarding data availability:

The datasets used and/or analyzed during the current study are available in the following link: <https://nihcc.app.box.com/v/ChestXray-NIHCC/>.

Apart from the original source of the dataset, it is also publicly accessible via kaggle. To access via Kaggle: <https://www.kaggle.com/nih-chest-xrays/data>.

**Dataset Pre-Processing and Implementation Details**

In its default version, the dataset consists of chest X-ray images in subfolders. In order to be used as input in deep learning architectures, some pre-processing steps were applied without disturbing the structure of the images. The chest X-ray images in the dataset were collected under a single folder and resized to 224x224. In this way, the images were made ready for use in training by ensuring that the images were in a single size.

if you want to access a pre-processed version of the dataset: <https://www.kaggle.com/datasets/khanfashee/nih-chest-x-ray-14-224x224-resized>.
